# Novel Antibiotics Targeting Bacterial Replicative DNA Polymerases

**DOI:** 10.3390/antibiotics9110776

**Published:** 2020-11-04

**Authors:** Joana A. Santos, Meindert H. Lamers

**Affiliations:** Department of Cell and Chemical Biology, Leiden University Medical Center, 2333 ZC Leiden, The Netherlands; j.a.santos@lumc.nl

**Keywords:** antibiotic discovery, DNA replication, DNA polymerase, *M. tuberculosis*

## Abstract

Multidrug resistance is a worldwide problem that is an increasing threat to global health. Therefore, the development of new antibiotics that inhibit novel targets is of great urgency. Some of the most successful antibiotics inhibit RNA transcription, RNA translation, and DNA replication. Transcription and translation are inhibited by directly targeting the RNA polymerase or ribosome, respectively. DNA replication, in contrast, is inhibited indirectly through targeting of DNA gyrases, and there are currently no antibiotics that inhibit DNA replication by directly targeting the replisome. This contrasts with antiviral therapies where the viral replicases are extensively targeted. In the last two decades there has been a steady increase in the number of compounds that target the bacterial replisome. In particular a variety of inhibitors of the bacterial replicative polymerases PolC and DnaE have been described, with one of the DNA polymerase inhibitors entering clinical trials for the first time. In this review we will discuss past and current work on inhibition of DNA replication, and the potential of bacterial DNA polymerase inhibitors in particular as attractive targets for a new generation of antibiotics.

## 1. Introduction

### 1.1. The Bacterial Replisome

**Figure 1:**
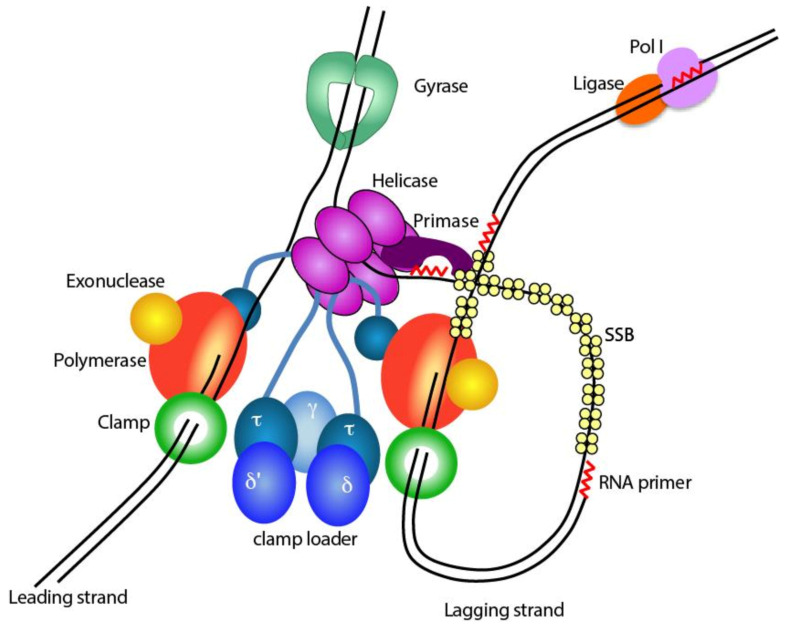
Schematic representation of the bacterial replisome. From top to bottom: DNA Gyrase removes positive supercoiling from the DNA that is induced by the unwinding of the two strands by the DNA Helicase. The helicase also recruits the primase that creates RNA primers for the discontinuous DNA synthesis on the lagging strand. Each of the two strands are replicated by a DNA polymerase that is bound to an exonuclease and a Clamp. The two polymerases remain connected via the clamp loader subunit τ that also binds to the helicase. The clamp loader complex comprising δ, δ′, τ and γ subunits loads the clamp onto the RNA primers created by the primase. Single stranded DNA binding protein (SSB) tetramers coat the single stranded DNA (ssDNA) on the lagging strand, while DNA Polymerase I (Pol I) and DNA Ligase remove the RNA primer and close the nicks.

Antibiotics can be broadly divided into four groups based on their target [1,2]: (i) those that target the cell membrane directly or inhibit membrane biosynthesis through a number of enzymes in the membrane biosynthesis pathway, (ii) those that inhibit protein synthesis by targeting the ribosome, (iii) those that inhibit RNA synthesis by targeting the RNA polymerase, and (iv) those that inhibit RNA and DNA synthesis by targeting DNA gyrases and topoisomerases. Yet, surprisingly, there are currently no antibiotics that directly target any of the DNA replisomal proteins, despite them being an attractive target for novel antibiotics. The replisome consists of a large number of subunits that work together in a well-orchestrated manner (Figure 1). Its extreme complexity makes it sensitive to perturbations, as shown by the large number of small molecule compounds that have been found to inhibit the different subunits of the replisome [3,4,5]. Furthermore, as every cell starts with replication of the genome, the inhibition of DNA replication is, quite literally, the most direct way to prevent the start of a new cell.

The complexity of the replisome has its origin in the two DNA strands that run in opposite polarity to each other [6]. DNA synthesis, however, only occurs in a single direction, from the 5′ end to 3′ end of the DNA strand. To ensure that both strands are replicated simultaneously, multiple proteins are required that assemble into the replisome [7]. Within the replisome, one strand is replicated in a continuous manner (leading strand), while the other strand is replicated in a discontinuous manner (lagging strand). This requires a large number of events that need to be carefully coordinated. The DNA helicase DnaB travels at the front of the replisome and separates the two DNA strands [8]. Then before the DNA polymerase can start DNA synthesis, two events need to occur: an RNA primer is synthesized by the RNA primase DnaG as the polymerase cannot start DNA synthesis de novo [9], and the DNA sliding clamp β is loaded onto the RNA primer to provide processivity to the DNA polymerase. Once attached to the β-clamp, the DNA polymerase can synthesize DNA in stretches of up 100,000 base pairs (bp) per single binding event at speeds of up to 1000 nucleotides per second (nt/s) [10,11]. The β-clamp is a circular shaped protein, that cannot load itself onto the DNA and is therefore loaded onto the DNA by a dedicated clamp loader complex consisting of the subunits δ, δ′, γ and τ, that opens the clamp and places it onto the RNA primer [12]. The τ subunit of the clamp loader furthermore binds to the DnaB helicase and to the leading and lagging strand polymerases, thus keeping all activities associated into one large complex [13,14].

While the leading strand, in theory, only needs a single loading event, the lagging strand requires thousands of repeated cycles of RNA priming, clamp loading and re-initiation of DNA synthesis [15,16]. With an average length of 500–1000 bp per fragment and an average genome size of 4.5 million bp in *E. coli*, the re-initiation of DNA synthesis occurs 4500 times per replication cycle. At speeds of up to a ~1000 nt/s; this implies that every second the cycle of priming, clamp loading and re-initiation of DNA synthesis is repeated [8,17]. Furthermore, on the lagging strand, large gaps of single stranded DNA (ssDNA) are created between the helicase and the polymerase, which are coated by the single stranded DNA binding protein (SSB) to protect the fragile ssDNA and prevent secondary structures that are an obstruction to the DNA polymerase [18]. Finally, after the whole genome has been replicated, the thousands of RNA primers on the lagging strand are removed and gaps sealed by the consorted action of DNA polymerase I and DNA ligase LigA [19].

Two additional proteins are required for efficient DNA replication: DNA gyrase and topoisomerase IV [20]. DNA gyrase releases the positive supercoils that accumulate ahead of the fork due to the unwinding of the two strands by the DNA helicase. Topoisomerase IV functions behind the replisome and relieves negative supercoiling of the DNA once it leaves the replisome.

Of the many potential targets in the replication, only gyrase and topoisomerase IV are common targets for antibiotics [21]. Although they execute opposite reactions (negative vs. positive supercoiling), they are closely related in structure, and consequently many antibiotics such as nalidixic acid, novobiocin, and ciprofloxacin target both gyrase and topoisomerase IV [22]. Encouragingly, in recent years, several other replisome proteins have been targeted by small molecules, including the DNA helicase DnaB, the DNA sliding clamp β, the RNA primase DnaG, DNA ligase LigA, as well as the replicative DNA polymerases PolC and DnaE [4,5,23]. In particular the DNA polymerases are of interest as they are structurally distinct from the eukaryotic DNA polymerases. They furthermore contain multiple potential sites that can be targeted as they contain both a polymerase and exonuclease active sites, and are dependent on its interaction with other replisomal proteins, such as, the DNA sliding clamp β and the clamp loader subunit τ. In this review, we will discuss past and current efforts on finding novel inhibitors of the bacterial DNA polymerases, as well as discuss directions for development of novel antibiotics that will be needed to combat the ever-increasing threat of multidrug-resistant bacteria.

### 1.2. The Bacterial Replicative DNA Polymerases

**Figure 2:**
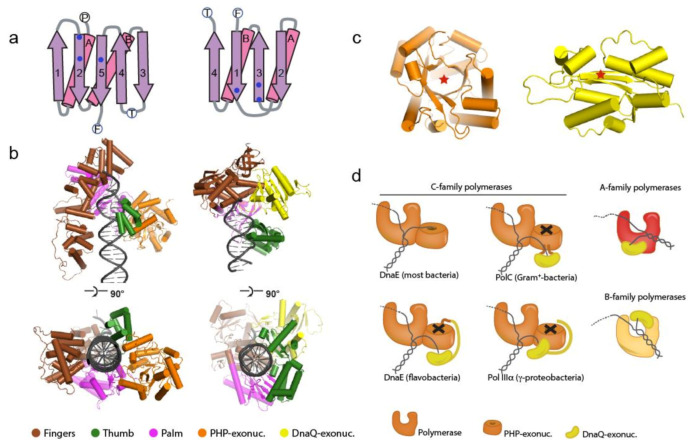
Unique features of bacterial replicative DNA polymerases. (**a**). The active site of the bacterial replicase consists of a 5-stranded parallel/antiparallel β-sheet. The active site of the eukaryotic replicative DNA polymerase contains 4-stranded, all antiparallel β-sheet. Catalytic site residues, marked with blue dots, are located at opposite site of the β-sheet—adapted from [28]. (**b**). Comparison of the bacterial replicative DNA polymerase (left, PDB: 5FKW, EMDB: 3202) and the eukaryotic replicative DNA polymerase δ (right, PDB: 3IAY). Domains are indicated with different colors. The C-terminal tail of bacterial polymerase is omitted for clarity. (**c**). The Polymerase and Histidinol Phosphatase (PHP)-exonuclease (left) and DnaQ-type exonuclease (right) are structurally distinct. The red star indicates the location of active site (**d**). Schematic representation of the different polymerase families, highlighting the different locations of the PHP- and DnaQ-exonuclease domains. Black cross marks inactive PHP-exonucleases.

All bacterial replicative DNA polymerases belong to the C-family of DNA polymerases, which are only found in the bacterial kingdom [24,25]. In contrast, the eukaryotic replicative DNA polymerases belong to the B-family polymerases [26]. The C-family polymerases are distinct from the B- family polymerases in that the palm domain, on which the three catalytic site residues are located, forms a five-stranded β-sheet with two parallel and three anti-parallel strands [27,28], while the palm domain of the B-family polymerase forms a four-stranded anti-parallel β-sheet (Figure 2a). In addition, the amino acid residues that form the catalytic triad lay on opposite sides of the β-sheet. The fingers domain of the C-family polymerases which interacts with the incoming template strand as well as the downstream DNA is ~2 times larger than that in B-family polymerases (Figure 2b), and is followed by a 100–150-residue C-terminal tail that interacts with the DNA sliding clamp β and the τ subunit of the clamp loader complex. Finally, the C-family DNA polymerases contain an N-terminal “Polymerase and Histidinol Phosphatase” (PHP) domain that is unique to bacteria, and a few isolated fungi [29] (Figure 2c). In the majority of bacteria, the PHP domain functions as the 3′-5′ exonuclease that removes mis-incorporated nucleotides from the growing newly synthesized strand [30]. In several bacterial species the PHP exonuclease has been superseded by a DnaQ-type of exonuclease [30,31], which is similar to the exonuclease domain found in the B-family DNA polymerases (Figure 2c). Based on the type and location of the 3′-5′ exonuclease domain, the C-family of DNA polymerases can be divided into separate classes. More than half of the C-type polymerases show an intact PHP domain and it was shown that in *M. tuberculosis* and *M. smegmatis* the PHP domain is the replicative 3′-5′ exonuclease that is essential for DNA replication and cell fitness [30]. Phylogenetic analysis suggests that in at least three separate events the role of the PHP-exonuclease was replaced by a DnaQ-type exonuclease domain [30,31]. In Gram-positive bacteria, which include several pathogenic bacteria such as *Enterococcus faecium*, *Staphylococcus aureus*, *Streptococcus pyogenes* and *Clostridium difficile*, the DnaQ-type exonuclease is inserted into the middle of the PHP domain, giving rise to the replicative DNA polymerase PolC. In the γ-proteobacteria that include the well-studied *E. coli*, as well as several pathogens such as the *Salmonella* species, *Vibrio cholerae* and *Yersinia pestis*, the PHP-exonuclease has been inactivated (but not removed) and replaced by a DnaQ-type exonuclease that is a separate subunit (ε) that binds tightly to the PHP domain of replicative polymerase Pol IIIα. Finally, in the class of flavobacteria, which contains no known human pathogens, the DnaQ domain is fused N-terminally to the PHP domain. Interestingly, in all three cases, the DnaQ-exonuclease associates with the PHP domain, while in the A- and B-family of polymerase (which do not contain a PHP domain), the DnaQ-type exonuclease is associate with the palm domain or fingers–thumb domains, respectively (Figure 2d). Hence the unique structural features of the C-family DNA polymerases, which comprise all bacterial replicative DNA polymerases, make it an attractive target for novel antibiotics that are essential to all bacterial forms of life, but distinct from their eukaryotic counterparts Pol ε and Pol δ.

## 2. Inhibiting Bacterial Replicative DNA Polymerases

### 2.1. Polymerase Active Site Inhibitors

One of the earliest and most commonly used modes of inhibition of a DNA polymerase is applied in Sanger sequencing [32] where the incorporation of a dideoxynucleotide leads to termination of DNA synthesis. The lack of the 3′ hydroxyl in the ribose of the dideoxynucleotide prevents the incorporation of the next nucleotide, which thus acts as a chain terminator. Nucleotide analogs that are used today (often administered as a pro-drug in the form of a nucleoside analog that is converted in the triphosphate nucleotide by the cell) work along the same principle. They are most successfully used in antiviral therapy [33], where they target either the viral DNA polymerases [34] (such as acyclovir for treatment of herpes virus), reverse transcriptases [35] (such as azidothymine (AZT) for treatment of HIV) and RNA dependent RNA polymerases (such as remdesivir that is currently being tested for treatment of SARS-CoV-2 virus) [36]. Nucleotide analogs are highly effective against viral reverse transcriptases and RNA dependent RNA polymerases as these do not contain a 3′-5′ exonuclease that can remove the incorporated nucleotide analog from the DNA. Viral DNA polymerases on the other hand, such as the UL30 gene product from herpes virus, do contain a 3′-5′ exonuclease, but here the lack of the ribose in acyclovir make it a poor substrate for the exonuclease [37] thus preventing removal from the newly synthesized strand and resulting in termination of DNA synthesis.

It was recently shown that nucleotide analogs may also have a potential to be effective in treatment of human cancers associated with mutations in the exonuclease domain of the replicative DNA polymerase Pol ε [38]. The mutations in the exonuclease domain render it inactive, resulting in a hypermutator phenotype that acts as a driver for the development of cancer [39]. Yet, the lack of exonuclease activity also renders these cancers highly sensitive to nucleotide analogs as the mutated exonuclease is no longer able to remove the nucleotide analog from the DNA, whereas the healthy, wild-type cells are able to remove the nucleotide analogs and are therefore resistant to the killing of the cell.

**Figure 3:**
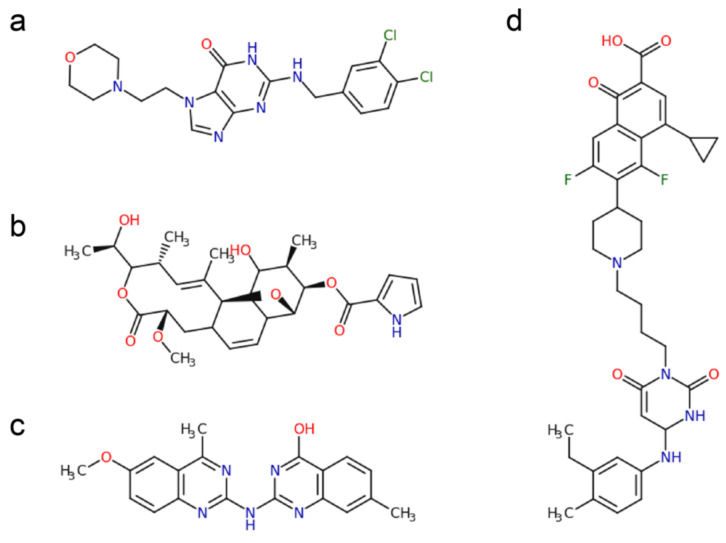
Chemical structures of a selection of bacterial DNA polymerase inhibitors. (**a**). ACX-362E [41] (**b**). Nargenicin [40]. (**c**). Quinazolin-2-ylamino-quinazolin-4-ol (compound 17) [47]. (**d**). 251D [48].

Remarkably, there are currently no nucleotide analogs used in the treatment of bacterial infections. Given the many differences between the bacterial and eukaryotic polymerases, it stands to reason that suitable analogs can be found that specifically target the bacterial replicative polymerases, but not the human polymerases. Indeed, several inhibitors of bacterial replicative DNA polymerases have been discovered in the last two decades (see Table 1). These inhibitors show a variety of chemical backbones (Figure 3), with both nucleoside and non-nucleoside analogs that target the polymerase active site, while others such as Nargenicin, appear to target a site distal from the polymerase active site [40]. Excitingly, the first bacterial specific polymerase inhibitor, Ibezapolstat (ACX-362E) that inhibits the *C. difficile* PolC [41] is currently in Phase 2 clinical trials. This inhibitor contains a guanine-like moiety that base-pairs with a cytosine in the DNA template strand, while the remaining part of the inhibitor molecule binds near to the deoxynucleoside triphosphates dNTPs binding site, rendering the polymerase inactive [41].

### 2.2. Allosteric Inhibition

In addition to directly targeting the catalytic site of the polymerase, there are also inhibitors that act away from the active site through allosteric inhibition [50]. Several non-nucleoside analogs used in antiviral therapies inhibit the HIV reverse transcriptase through binding of a non-catalytic pocket 10 Å away from the polymerase active site [51,52]. Similarly, the recently discovered natural compound Nargenicin, isolated from the bacterium *Nocardia devorans*, appears to target a non-catalytic site. Nargenicin was found to be potent inhibitor of the DnaE polymerase from the Gram-positive bacterium *Staphylococcus aureus* [40]. A resistant mutation Serine 765 to Leucine, equivalent to residue 834 in *E. coli* Pol IIIα is located close to the exit path of the DNA, 30 Å away from the polymerase active site, when mapped onto the crystal structure of Pol IIIα [28].

DNA polymerases may also present other sites of inhibition, as they are dynamic proteins that need their mobility for their activity. For example, in *E. coli* Pol IIIα, the C-terminal tail is moving ~70 Å between DNA-bound state and DNA free state, a movement that has been suggested to act as a molecular switch that aids in the repeated release and rebinding of the lagging strand polymerase [53]. Furthermore, during transfer from the polymerase to exonuclease active site, the primer strands passes the thumb domain of the polymerase that moves away, while the exonuclease domain moves towards the primers strand [54]. Hence, compounds that restrict these various movements in the polymerase are likely to result in inhibition of the polymerase.

### 2.3. Exonuclease Inhibitors

All replicative DNA polymerases contain a 3′-5′ exonuclease that is essential for high-fidelity DNA replication [55,56]. They are often referred to as proofreaders, an unfortunate term as the proofreading of incorporated nucleotides occurs in the polymerase active site. The incorporation of the wrong nucleotide leads to stalling of the polymerase activity, reduced affinity for the DNA substrate and the transfer of the newly synthesized strand to the exonuclease active site [57]. The exonuclease itself indiscriminately removes the last incorporated nucleotide from the 3′ end of the DNA [58,59]. A more suitable term for the replicative 3′-5′ exonuclease is therefore “roadblock remover” as it removes mis-incorporated nucleotides and modified nucleotides that form a block to the replicative DNA polymerase. Consequently, when exonuclease activity is deleted in *E. coli*, *Salmonella typhimurium*, or *M. tuberculosis*, the cells become compromised with strongly reduced growth rates and increased mutation rates [30,60,61].

Inhibitors of the exonuclease can either act through incorporation of a nucleotide analog into the newly synthesized strand that cannot be removed, such as antiviral agent acyclovir [62] (Table 2). The exonuclease active site can also be directly targeted with small molecules, as exemplified by the *p*-nitrophenyl ester of thymidine 5′-monophosphate (pNP-TMP) that can be used to monitor exonuclease activity [63]. Although it is readily hydrolyzed by the exonuclease, it illustrates that small molecules can target the exo site, and suggests that in a non-hydrolysable form could be an inhibitor. Indeed, a fascioquinol was found to be a potent inhibitor of the *Streptococcus pneumoniae* PolC exonuclease [64]. Small molecule-based inhibition of PHP-exonucleases might be even more successful as their active site consists of a deep pocket [65] that is well suited for binding of inhibitors. In agreement, a recent study with a select number of nucleotides found that the dinucleotides dADP, dCDP and dGDP inhibit the exonuclease activity of the *M. tuberculosis* PHP-exonuclease [66].

Inhibition of the replicative exonuclease can be further potentiated through the addition of nucleotide analogs that target the polymerase. A proof of concept was shown by Rock et al., where an inactive PHP-exonuclease mutant of *M. smegmatis* DnaE1 rendered cells susceptible to the nucleotide analog Ara-A (also known as vidarabine, previously used in treatment of herpes virus), while wild-type cells remained unaffected [30]. As a significant part of tuberculosis patients in the southern half of Africa are co-infected with HIV, they often receive nucleoside analogs for the treatment of AIDS [67]. Co-administration of a *M. tuberculosis*-specific exonuclease inhibitor in combination with a nucleoside analog-based treatment of HIV could therefore act synergistically, treating both HIV and *M. tuberculosis* simultaneously.

### 2.4. Disrupting Interaction with Replisome Proteins

**Figure 4:**
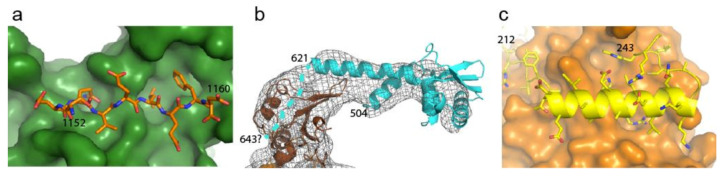
Protein–protein interaction sites in the *E. coli* replicative DNA polymerase Pol IIIα. (**a**). The β-clamp binding pocket bound by the Pol IIIα peptide. β-clamp in green, Pol IIIα peptide in orange sticks (PDB: 3D1F) [70]. (**b**). Cryogenic electron-microscopy (Cryo-EM) map showing Pol IIIα–τ interaction. C-terminal tail of Pol IIIα in brown, C-terminal tail of τ shown in cyan (PDB: 5FKV, EMDB: 3198) [53]. The potential position of the last ~20 residues of τ are indicated with a blue dashed line. (**c**). DnaQ-type exonuclease binding to the PHP domain of Pol IIIα (PDB: 4GX8) [76]. PHP domain in orange surface, tail of exonuclease in yellow cartoon and sticks. Numbers indicate amino acid residue number.

In isolation, the *E. coli* Pol IIIα polymerase is a poor enzyme but once assembled into the replisome it reaches speeds of 1000 nt/s and 100,000 bp synthesized in a single binding event [8,68]. Within the replisome, Pol IIIα directly interacts with the DNA sliding clamp β (Figure 4a) that provides processivity to the polymerase. Work by Wijffels et al. showed that peptides derived from β-binding protein could disrupt the interaction between *E. coli* Pol IIIα and the β-clamp [69] (Table 3). Similarly, a targeted screen of a 30,000 small-compound library revealed several compounds that target the β-clamp binding pocket and inhibit the interaction with the replicative polymerase Pol IIIα [70]. Finally, the natural compound Griselimycin derived from *Streptomyces*, is a potent inhibitor of *M. tuberculosis*, and was found to bind to the canonical binding pocket of the β-clamp [71].

The polymerase further interacts with the clamp loader subunit τ that couples both leading and lagging strand polymerase into one complex [72]. The interaction between Pol IIIα and τ is very tight [73] and prevents the polymerases from leaving the replisome during DNA replication [10]. The interaction occurs via the last, unstructured ~20 amino acids of τ [53,74] (Figure 4b), suggesting it binds to a bespoke binding pocket in the polymerase, which could be potentially targeted by various compounds.

Finally, in γ-proteobacteria, which include *E. coli*, *Salmonella typhimurium*, and *Yersinia pestis*, the exonuclease subunit is not an integral part of the polymerase but is provided for by a separate subunit ε, that binds to the polymerase with high affinity [75]. The ε subunit binds to the polymerase PHP domain through a helix that presents several residues that are buried in a pocket of the PHP domain [76] (Figure 4c) that could be targeted by small compounds.

### 2.5. Targeting DNA Polymerases to Combat Antibiotic Resistance

Bacteria employ several mechanisms to drive resistance to antibiotics, including increased clearance by membrane pumps, enzymatic inactivation of the antibiotic, or mutation of the target site to reduce affinity of the inhibitor [78]. Mutations in turn can be generated by a number of pathways including DNA replication [79], DNA repair, or through the action of error-prone polymerases [80]. In particular, mycobacteria, including *M. tuberculosis*, contain a second copy of the replicative DNA polymerase: DnaE2. Under optimal growth conditions, DnaE2 is not detectable but is expressed upon exposure to antibiotics or genotoxic stress [81]. Expression of DnaE2 correlates with increased mutations rates and antibiotic resistance. Reversely, deletion of the *dnaE2* gene results in a reduction in drug resistance in culture and in mice. Importantly, the PHP-exonuclease domain of DnaE2 lacks a critical conserved residue in its active site that renders the exonuclease inactive [66], which makes this polymerase an ideal target for nucleotide analogs that through their inhibition of this error prone polymerase may be able to reduce the mutation rate and emergence of antibiotic resistance in *M. tuberculosis*.

## 3. High-Throughput Screening Methods for Replication Inhibitors

DNA replication has been studied for several decades and multiple assays have been developed to monitor the activity of the polymerase and exonuclease. Gel-based assays are very informative on the behavior of polymerase and can elucidate at what point and how inhibition takes place. Yet, these are less suited for high-throughput analysis of large libraries of compounds. Instead several fluorescence-based assays are much better suited for high-throughput screening campaigns.

Bacteriophage derived ssDNA and the dsDNA-specific fluorescent dye PicoGreen that strongly increases fluorescence upon dsDNA binding were used to discover multiple inhibitors of the *E. coli* and *B. subtilis* replisomes [3]. Smaller DNA substrates in contrast are more suitable for individual polymerase or exonuclease targeted assays. For example, an assay described by Song et al. makes use of the quenching properties of guanines. In this assay, a fluorescent dye is attached to the 5′ end of the template strand that ends with a stretch of cytidines. When the polymerase extends the primer strand and incorporates multiple deoxyguanosine monophosphates (dGMPs) into the new strand, this will result in a reduction of fluorescence intensity [82]. Reversely, by initiating the reaction with a fully extended primer strand, one can monitor exonuclease activity, as the removal of the dGMPs in the primer strand will result in an increase in fluorescence intensity [83]. An alternative assay was developed by Shapiro et al., which uses a donor–acceptor fluorophore pair to measure the stability of the primer–template duplex by means of Förster resonance energy transfer (FRET). The length of the primer is chosen such that addition of 4 M urea results in melting of the duplex and consequent decrease in the FRET signal. However, the extension of the primer strand by a DNA polymerase increases the stability of the DNA duplex, which no longer dissociates upon addition of 4 M urea [84]. Finally, the use of the non-canonical nucleoside analog pNP-TMP, an ester fusion of a phenol group and TMP can be used to monitor the activity of the exonuclease [63].

To discover inhibitors of the interaction between polymerase and its replisomal partners β, τ, and ε, assays based on fluorescent peptides are well suited for high-throughput screening. For example, such an approach was used to discover inhibitors of the *E. coli* Pol IIIα- β interaction [70]. The same approach will be applicable to find inhibitors of the binding between the polymerase and τ and in the γ-proteobacteria, between the polymerase and the exonuclease ε.

## 4. Conclusions

DNA replication is an essential process for the maintenance of life and relies on the carefully organized action of a large number of proteins. Consequently, even mild disturbances in its dynamics could potentially lead to inhibition of DNA replication and termination of cell growth. However, replisomal proteins, including the replicative DNA polymerases, have thus far not been used as targets in antibacterial regiments. This is surprising when considering that bacterial replicative DNA polymerases are different in both sequence and structure from the human replicative DNA polymerases. The major structural assets of bacterial replicative polymerases that discriminate them from their eukaryotic counterparts are also those assets that make them attractive targets for antibiotics design and include: (i) a structurally distinct active site with a different location within the palm domain; (ii) a larger fingers domain that is the interaction site for the replisomal proteins β and τ and (iii) the unique PHP-type exonuclease domain, that is only found in bacteria and is structurally distinct from the human replicative exonucleases.

The last two decades have yielded numerous examples of compounds that selectively inhibit the bacterial replicative DNA polymerase. However, the process of developing an early hit compound into an effective novel antibiotic is riddled with hurdles and consequently it is no surprise that only one polymerase inhibitor so far has made it to the clinical stage.

However, with the tools at hand for high-throughput screening and detailed knowledge about the functioning of the bacterial replicative DNA polymerases and their essential interactions with other replisomal proteins, it will be possible to find many novel inhibitors that would be suitable candidates for a hit-to-lead campaign and ultimately the development of novel antibiotics that are desperately needed in the continuing fight against multi-drug resistant bacteria.

## Figures and Tables

**Table 1:** Bacterial DNA polymerase inhibitors.

Compound	Nucleotide	Polymerase	Species	Reference
**ACX-362E ^1^**	Yes	PolC	*C. difficile*	[41]
**ACX-375C**	Yes	PolC	*S. aureus, others*	Acurxpharma ^3^
**Nargenicin**	No	DnaE	*S. aureus*	[40]
**DB04118, DB04698 ^2^**	No	Pol IIIα	*E. coli*	[42]
**NBH**	No	PolC	*S. aureus*	[43]
**Multiple ^2^**	Yes, No	DnaE2 ^4^	*M. tuberculosis*	[44]
**324C**	Yes	DnaE	*B. subtilis*	[45]
**7-Alkyl-N(2)-substituted-3-deazaguanines**	Yes	PolC, Pol IIIα	*B. subtilis*	[46]
**quinazolin-2-ylamino-quinazolin-4-ols**	No	PolC	*S. aureus*	[47]
**251D**	No	PolC	*B. subtilis*	[48]
**6-Anilinouracil-based**	Yes	PolC	*B. subtilis*	[49]

^1^ Ibezapolstat, the first bacterial polymerase inhibitor advanced to the clinic. ^2^ in silico screen only. ^3^ https://www.acurxpharma.com/pipeline/acx-375c. ^4^ DnaE2 is a non-replicative DNA polymerase involved in drug resistance in M. tuberculosis.

**Table 2:** Bacterial replicative exonuclease inhibitors.

Compound	Target Site	Polymerase	Species	Reference
**dADP, dCDP, dGDP**	PHP	DnaE1	*M. tuberculosis*	[66]
**fascioquinol**	exo-PHP	PolC	*S. pneumoniae*	[64]

**Table 3:** Disruptors of polymerase interaction.

Compound	Target Site	Polymerase	Species	Reference
**Griselimycin**	clamp	DnaE1	*M. tuberculosis*	[71]
**Vedaprofen, bromfenac and carprofen**	clamp	Pol IIIα	*E. coli*, *S. aureus*, *B. subtilis*	[77]
**RU7 ^1^**	clamp	Pol IIIα	*E. coli*	[70]
**Peptides**	clamp	Pol IIIα	*E. coli*	[69]

^1^ thioxothiazolinine derivative.

## References

[1] Bbosa G.S., Mwebaza N., Odda J., Kyegombe D.B., Ntale M. (2014). Antibiotics/antibacterial drug use, their marketing and promotion during the post-antibiotic golden age and their role in emergence of bacterial resistance. Health.

[2] Kapoor G., Saigal S., Elongavan A. (2017). Action and resistance mechanisms of antibiotics: A guide for clinicians. J. Anaesthesiol. Clin. Pharmacol..

[3] Dallmann H.G., Fackelmayer O.J., Tomer G., Chen J., Wiktor-Becker A., Ferrara T., Pope C., Oliveira M.T., Burgers P.M.J., Kaguni L.S. (2010). Parallel Multiplicative Target Screening against Divergent Bacterial Replicases: Identification of Specific Inhibitors with Broad Spectrum Potential. Biochemistry.

[4] Reiche M.A., Warner D.F., Mizrahi V. (2017). Targeting DNA Replication and Repair for the Development of Novel Therapeutics against Tuberculosis. Front. Mol. Biosci..

[5] Causer R.J., Dixon N.E. (2012). Architecture and Conservation of the Bacterial DNA Replication Machinery, an Underexploited Drug Target. Curr. Drug Targets.

[6] O’Donnell M.E., Langston L., Stillman B. (2013). Principles and Concepts of DNA Replication in Bacteria, Archaea, and Eukarya. Cold Spring Harb. Perspect. Biol..

[7] Johnson A., O’Donnell M. (2005). Cellular DNA Replicases: Components and Dynamics at the Replication Fork. Annu. Rev. Biochem..

[8] Mok M., Marians K.J. (1987). The Escherichia coli preprimosome and DNA B helicase can form replication forks that move at the same rate. J. Biol. Chem..

[9] Wu A.C., Zechner E.L., Marians K.J. (1992). Coordinated leading- and lagging-strand synthesis at the Escherichia coli DNA replication fork. I. Multiple effectors act to modulate Okazaki fragment size. J. Biol. Chem..

[10] Lewis J.S., Spenkelink L.M., Jergic S., Wood E.A., Monachino E., Horan N.P., Duderstadt K.E., Cox M.M., Robinson A., Dixon N.E. (2017). Single-molecule visualization of fast polymerase turnover in the bacterial replisome. eLife.

[11] Tanner A.N., Hamdan S.M., Jergic S., Loscha K.V., Schaeffer P.M., Dixon N.E., Van Oijen A.M. (2008). Single-molecule studies of fork dynamics in Escherichia coli DNA replication. Nat. Struct. Mol. Biol..

[12] Stukenberg P.T., Studwell-Vaughan P.S., O’Donnell M. (1991). Mechanism of the sliding beta-clamp of DNA polymerase III holoenzyme. J. Biol. Chem..

[13] McHenry C.S. (1982). Purification and characterization of DNA polymerase III’. Identification of tau as a subunit of the DNA polymerase III holoenzyme. J. Biol. Chem..

[14] Onrust R., Finkelstein J., Turner J., Naktinis V., O’Donnell M. (1995). Assembly of a chromosomal replication machine: Two DNA polymerases, a clamp loader, and sliding clamps in one holoenzyme particle. III. Interface between two polymerases and the clamp loader. J. Biol. Chem..

[15] O’Donnell M.E. (1987). Accessory proteins bind a primed template and mediate rapid cycling of DNA polymerase III holoenzyme from Escherichia coli. J. Biol. Chem..

[16] Stukenberg P.T., Turner J., O’Donnell M. (1994). An explanation for lagging strand replication: Polymerase hopping among DNA sliding clamps. Cell.

[17] McInerney P., Johnson A., Katz F., O’Donnell M. (2007). Characterization of a Triple DNA Polymerase Replisome. Mol. Cell.

[18] Robinson A., Van Oijen A.M. (2013). Bacterial replication, transcription and translation: Mechanistic insights from single-molecule biochemical studies. Nat. Rev. Genet..

[19] Yang Y., Licata V.J. (2018). Pol I DNA polymerases stimulate DNA end-joining by Escherichia coli DNA ligase. Biochem. Biophys. Res. Commun..

[20] Sissi C., Palumbo M. (2010). In front of and behind the replication fork: Bacterial type IIA topoisomerases. Cell. Mol. Life Sci..

[21] Sanyal G., Doig P. (2012). Bacterial DNA replication enzymes as targets for antibacterial drug discovery. Expert Opin. Drug Discov..

[22] Pham T.D.M., Ziora Z.M., Blaskovich M.A.T. (2019). Quinolone antibiotics. MedChemComm.

[23] Kaguni J.M. (2018). The Macromolecular Machines that Duplicate the Escherichia coli Chromosome as Targets for Drug Discovery. Antibiotics.

[24] Ito J., Braithwaite D.K. (1991). Compilation and alignment of DNA polymerase sequences. Nucleic Acids Res..

[25] Delarue M., Poch O., Tordo N., Moras D., Argos P. (1990). An attempt to unify the structure of polymerases. Protein Eng. Des. Sel..

[26] Makarova K.S., Koonin E.V. (2013). Archaeology of Eukaryotic DNA Replication. Cold Spring Harb. Perspect. Biol..

[27] Bailey S., Wing R.A., Steitz T.A. (2006). The Structure of T. aquaticus DNA Polymerase III is Distinct from Eukaryotic Replicative DNA Polymerases. Cell.

[28] Lamers M.H., Georgescu R.E., Lee S.-G., O’Donnell M., Kuriyan J. (2006). Crystal Structure of the Catalytic α Subunit of E. coli Replicative DNA Polymerase III. Cell.

[29] Aravind L., Koonin E.V. (1998). Phosphoesterase domains associated with DNA polymerases of diverse origins. Nucleic Acids Res..

[30] Rock J.M., Lang U.F., Chase M.R., Ford C.B., Gerrick E.R., Gawande R., Coscolla M., Gagneux S., Fortune S.M., Lamers M.H. (2015). DNA replication fidelity in Mycobacterium tuberculosis is mediated by an ancestral prokaryotic proofreader. Nat. Genet..

[31] Timinskas K., Balvočiūtė M., Timinskas A., Venclovas Č. (2013). Comprehensive analysis of DNA polymerase III α subunits and their homologs in bacterial genomes. Nucleic Acids Res..

[32] Sanger F., Nicklen S., Coulson A.R. (1977). DNA sequencing with chain-terminating inhibitors. Proc. Natl. Acad. Sci. USA.

[33] Jordheim L.P., Durantel D., Zoulim F., Dumontet C. (2013). Advances in the development of nucleoside and nucleotide analogues for cancer and viral diseases. Nat. Rev. Drug Discov..

[34] Zarrouk K., Piret J., Boivin G. (2017). Herpesvirus DNA polymerases: Structures, functions and inhibitors. Virus Res..

[35] Fischl M.A., Richman D.D., Grieco M.H., Gottlieb M.S., Volberding P.A., Laskin O.L., Leedom J.M., Groopman J.E., Mildvan D., Schooley R.T. (1987). The Efficacy of Azidothymidine (AZT) in the Treatment of Patients with AIDS and AIDS-Related Complex. N. Engl. J. Med..

[36] Tempestilli M., Caputi P., Avataneo V., Notari S., Forini O., Scorzolini L., Marchioni L., Bartoli T.A., Castilletti C., Lalle E. (2020). Pharmacokinetics of remdesivir and GS-441524 in two critically ill patients who recovered from COVID-19. J. Antimicrob. Chemother..

[37] Vashishtha A.K., Kuchta R.D. (2016). Effects of Acyclovir, Foscarnet, and Ribonucleotides on Herpes Simplex Virus-1 DNA Polymerase: Mechanistic Insights and a Novel Mechanism for Preventing Stable Incorporation of Ribonucleotides into DNA. Biochemistry.

[38] Van Gool I.C., Rayner E., Osse E.M., Nout R.A., Creutzberg C.L., Tomlinson I., Church D.N., Smit V.T., De Wind N., Bosse T. (2018). Adjuvant Treatment forPOLEProofreading Domain–Mutant Cancers: Sensitivity to Radiotherapy, Chemotherapy, and Nucleoside Analogues. Clin. Cancer Res..

[39] Campbell B.B., Light N., Fabrizio D., Zatzman M., Fuligni F., De Borja R., Davidson S., Edwards M., Elvin J.A., Hodel K.P. (2017). Comprehensive Analysis of Hypermutation in Human Cancer. Cell.

[40] Painter R.E., Adam G.C., Arocho M., DiNunzio E., Donald R.G., Dorso K., Genilloud O., Gill C., Goetz M., Hairston N.N. (2015). Elucidation of DnaE as the Antibacterial Target of the Natural Product, Nargenicin. Chem. Biol..

[41] Xu W.-C., Silverman M.H., Yu X.Y., Wright G., Brown N. (2019). Discovery and development of DNA polymerase IIIC inhibitors to treat Gram-positive infections. Bioorganic Med. Chem..

[42] Mondal S.I., Ferdous S., Akter A., Mahmud Z., Karim N., Islam M.S., Jewel N.A., Afrin T. (2015). Identification of potential drug targets by subtractive genome analysis of Escherichia coli O157:H7: An in silico approach. Adv. Appl. Bioinform. Chem..

[43] Hou Z., Zhou Y., Li J., Zhang X., Shi X., Xue X., Li Z., Ma B., Wang Y., Li M. (2015). Selective in vivo and in vitro activities of 3,3′-4-nitrobenzylidene-bis-4-hydroxycoumarin against methicillin-resistant Staphylococcus aureus by inhibition of DNA polymerase III. Sci. Rep..

[44] Jadaun A., Sudhakar D.R., Subbarao N., Dixit A. (2015). In silico screening for novel inhibitors of DNA polymerase III alpha subunit of Mycobacterium tuberculosis (MtbDnaE2, H37Rv). PLoS ONE.

[45] Barnes M.H., Butler M.M., Wright G.E., Brown N.C. (2012). Antimicrobials targeted to the replication-specific DNA polymerases of gram-positive bacteria: Target potential of dnaE. Infect. Disord. Drug Targets.

[46] Xu W.-C., Wright G.E., Brown N.C., Long Z.-Y., Zhi C.-X., Dvoskin S., Gambino J.J., Barnes M.H., Butler M.M. (2011). 7-Alkyl-N(2)-substituted-3-deazaguanines. Synthesis, DNA polymerase III inhibition and antibacterial activity. Bioorganic Med. Chem. Lett..

[47] Guiles J.W., Sun X., Critchley I.A., Ochsner U., Tregay M., Stone K., Bertino J., Green L., Sabin R., Dean F.B. (2009). Quinazolin-2-ylamino-quinazolin-4-ols as novel non-nucleoside inhibitors of bacterial DNA polymerase III. Bioorganic Med. Chem. Lett..

[48] Butler M.M., LaMarr W.A., Foster K.A., Barnes M.H., Skow D.J., Lyden P.T., Kustigian L.M., Zhi C., Brown N.C., Wright G.E. (2007). Antibacterial Activity and Mechanism of Action of a Novel Anilinouracil-Fluoroquinolone Hybrid Compound. Antimicrob. Agents Chemother..

[49] Tarantino P.M., Zhi C., Gambino J.J., Wright G.E., Brown N.C. (1999). 6-Anilinouracil-Based Inhibitors of Bacillus subtilis DNA Polymerase III: Antipolymerase and Antimicrobial Structure−Activity Relationships Based on Substitution at Uracil N3. J. Med. Chem..

[50] Corona A., Masaoka T., Tocco G., Tramontano E., Le Grice S.F.J. (2013). Active site and allosteric inhibitors of the ribonuclease H activity of HIV reverse transcriptase. Futur. Med. Chem..

[51] Kohlstaedt A.L., Wang J., Friedman J.M., Rice P.A., Steitz T.A. (1992). Crystal structure at 3.5 A resolution of HIV-1 reverse transcriptase complexed with an inhibitor. Science.

[52] Ren J., Bird L., Chamberlain P.P., Stewart-Jones G.B., Stuart D.I., Stammers D.K. (2002). Structure of HIV-2 reverse transcriptase at 2.35-A resolution and the mechanism of resistance to non-nucleoside inhibitors. Proc. Natl. Acad. Sci. USA.

[53] Fernandez-Leiro R., Conrad J., Scheres S.H.W., Lamers M.H. (2015). Cryo-EM structures of the E. coli replicative DNA polymerase reveal its dynamic interactions with the DNA sliding clamp, exonuclease and τ. eLife.

[54] Fernandez-Leiro R., Conrad J., Yang J.-C., Freund S.M.V., Scheres S.H.W., Lamers M.H. (2017). Self-correcting mismatches during high-fidelity DNA replication. Nat. Struct. Mol. Biol..

[55] Bębenek A., Ziuzia-Graczyk I. (2018). Fidelity of DNA replication—A matter of proofreading. Curr. Genet..

[56] Brutlag D., Kornberg A. (1972). Enzymatic synthesis of deoxyribonucleic acid. 36. A proofreading function for the 3′ leads to 5′ exonuclease activity in deoxyribonucleic acid polymerases. J. Biol. Chem..

[57] Reha-Krantz L.J., Marquez-Curtis L., Elisseeva E., Baker R.P., Bloom L.B., Dunford H.B., Goodman M.F. (1998). The Proofreading Pathway of Bacteriophage T4 DNA Polymerase. J. Biol. Chem..

[58] Baker R.P., Reha-Krantz L.J. (1998). Identification of a transient excision intermediate at the crossroads between DNA polymerase extension and proofreading pathways. Proc. Natl. Acad. Sci. USA.

[59] Da Silva E.F., Reha-Krantz L.J. (2007). DNA polymerase proofreading: Active site switching catalyzed by the bacteriophage T4 DNA polymerase. Nucleic Acids Res..

[60] Lancy E.D., Lifsics M.R., Kehres D.G., Maurer R. (1989). Isolation and characterization of mutants with deletions in dnaQ, the gene for the editing subunit of DNA polymerase III in Salmonella typhimurium. J. Bacteriol..

[61] Scheuermann R., Tam S., Burgers P.M., Lu C., Echols H. (1983). Identification of the epsilon-subunit of Escherichia coli DNA polymerase III holoenzyme as the dnaQ gene product: A fidelity subunit for DNA replication. Proc. Natl. Acad. Sci. USA.

[62] Reardon E.J., Spector T. (1989). Herpes simplex virus type 1 DNA polymerase. Mechanism of inhibition by acyclovir triphosphate. J. Biol. Chem..

[63] Hamdan S., Bulloch E.M., Thompson P.R., Beck J.L., Yang J.Y., Crowther J.A., Lilley P.E., Carr P.D., Ollis D.L., Brown S.E. (2002). Hydrolysis of the 5′-p-nitrophenyl ester of TMP by the proofreading exonuclease (epsilon) subunit of Escherichia coli DNA polymerase III. Biochemistry.

[64] Standish A.J., Salim A.A., Capon R.J., Morona R. (2013). Dual inhibition of DNA polymerase PolC and protein tyrosine phosphatase CpsB uncovers a novel antibiotic target. Biochem. Biophys. Res. Commun..

[65] Banos-Mateos S., Van Roon A.-M.M., Lang U.F., Maslen S.L., Skehel J.M., Lamers M.H. (2017). High-fidelity DNA replication in Mycobacterium tuberculosis relies on a trinuclear zinc center. Nat. Commun..

[66] Nasir N., Kisker C. (2019). Mechanistic insights into the enzymatic activity and inhibition of the replicative polymerase exonuclease domain from Mycobacterium tuberculosis. DNA Repair.

[67] Cihlar T., Fordyce M. (2016). Current status and prospects of HIV treatment. Curr. Opin. Virol..

[68] Yao N.Y., Georgescu R.E., Finkelstein J., O’Donnell M.E. (2009). Single-molecule analysis reveals that the lagging strand increases replisome processivity but slows replication fork progression. Proc. Natl. Acad. Sci. USA.

[69] Wijffels G., Dalrymple B.P., Prosselkov P., Kongsuwan K., Epa V.C., Lilley P.E., Jergic S., Buchardt J., Brown S.E., Alewood P.F. (2004). Inhibition of protein interactions with the beta 2 sliding clamp of Escherichia coli DNA polymerase III by peptides from beta 2-binding proteins. Biochemistry.

[70] Georgescu R.E., Yurieva O., Kim S.-S., Kuriyan J., Kong X.-P., O’Donnell M. (2008). Structure of a small-molecule inhibitor of a DNA polymerase sliding clamp. Proc. Natl. Acad. Sci. USA.

[71] Kling A., Lukat P., Almeida D.V., Bauer A., Fontaine E., Sordello S., Zaburannyi N., Herrmann J., Wenzel S.C., König C. (2015). Targeting DnaN for tuberculosis therapy using novel griselimycins. Science.

[72] Duderstadt K.E., Geertsema H.J., Stratmann S.A., Punter C.M., Kulczyk A.W., Richardson C.C., Van Oijen A.M. (2016). Simultaneous Real-Time Imaging of Leading and Lagging Strand Synthesis Reveals the Coordination Dynamics of Single Replisomes. Mol. Cell.

[73] Jergic S., Ozawa K., Williams N.K., Su X.C., Scott D.D., Hamdan S.M., Crowther J.A., Otting G., Dixon N.E. (2007). The unstructured C-terminus of the tau subunit of Escherichia coli DNA polymerase III holoenzyme is the site of interaction with the alpha subunit. Nucleic Acids Res..

[74] Su X.C., Jergic S., Keniry M.A., Dixon N.E., Otting G. (2007). Solution structure of Domains IVa and V of the tau subunit of Escherichia coli DNA polymerase III and interaction with the alpha subunit. Nucleic Acids Res..

[75] Perrino F.W., Harvey S., McNeill S.M. (1999). Two functional domains of the epsilon subunit of DNA polymerase III. Biochemistry.

[76] Ozawa K., Horan N.P., Robinson A., Yagi H., Hill F.R., Jergic S., Xu Z.-Q., Loscha K.V., Li N., Tehei M. (2013). Proofreading exonuclease on a tether: The complex between the E. coli DNA polymerase III subunits α, ε, θ and β reveals a highly flexible arrangement of the proofreading domain. Nucleic Acids Res..

[77] Yin Z., Wang Y., Whittell L.R., Jergic S., Liu M., Harry E., Dixon N.E., Kelso M.J., Beck J.L., Oakley A.J. (2014). DNA Replication Is the Target for the Antibacterial Effects of Nonsteroidal Anti-Inflammatory Drugs. Chem. Biol..

[78] Reygaert W.C. (2018). An overview of the antimicrobial resistance mechanisms of bacteria. AIMS Microbiol..

[79] Warner D.F., Rock J.M., Fortune S.M., Mizrahi V. (2017). DNA Replication Fidelity in the Mycobacterium tuberculosis Complex. Adv. Exp. Med. Biol..

[80] Sutton M.D., Walker G.C. (2001). Managing DNA polymerases: Coordinating DNA replication, DNA repair, and DNA recombination. Proc. Natl. Acad. Sci. USA.

[81] Boshoff H.I., Reed M.B., Barry C.E., Mizrahi V. (2003). DnaE2 Polymerase Contributes to In Vivo Survival and the Emergence of Drug Resistance in Mycobacterium tuberculosis. Cell.

[82] Song C., Zhang C., Zhao M. (2009). Singly labeled smart probes for real-time monitoring of the kinetics of dNTP misincorporation and single nucleotide extension in DNA intra-molecular polymerization. Biosens. Bioelectron..

[83] Rêgo A.T., Holding A.N., Kent H., Lamers M.H. (2013). Architecture of the Pol III–clamp–exonuclease complex reveals key roles of the exonuclease subunit in processive DNA synthesis and repair. EMBO J..

[84] Shapiro A.B., Rivin O., Gao N., Hajec L. (2005). A homogeneous, high-throughput fluorescence resonance energy transfer-based DNA polymerase assay. Anal. Biochem..

